# Prevalence and Factors of Self-medication with Antibiotics in Claiborne County, Tennessee

**DOI:** 10.13023/jah.0501.05

**Published:** 2023-04-01

**Authors:** Kimberly A. Carney, Lauren Wisnieski, Tristan Lackey, Donald Noah

**Affiliations:** Lincoln Memorial University-College of Veterinary Medicine, kimberly.carney@lmunet.edu; Lincoln Memorial University-College of Veterinary Medicine; Lincoln Memorial University-College of Veterinary Medicine; Lincoln Memorial University-College of Veterinary Medicine

**Keywords:** Appalachia, antimicrobial resistance, antibiotic resistance, self-medication

## Abstract

**Introduction:**

Antimicrobial resistance (AMR) is a serious concern to public health, causing an estimated 35,000 deaths annually in the U.S. Misuse of antimicrobials increases the rate of AMR. Self-medication with antibiotics (SMA) is a primary contributor to AMR that can be addressed through education. SMA has been reported at rates of 3% to 66% in the U.S. but has not been evaluated in Appalachia.^1^ Low health literacy and barriers to accessing care have been correlated with SMA and are common in many areas of Appalachia.

**Purpose:**

This study aims to assess factors associated with SMA, demographic differences in knowledge of / beliefs about SMA, and describe practices and beliefs of those who self-medicate in the Cumberland Gap region.

**Methods:**

Structured interviews were conducted in a rural health clinic and in a dental office to ascertain demographic information, knowledge of appropriate antibiotic use, and behaviors associated with self-medication. Inferential statistics (chi-squared, Fisher’s exact, and ANOVA tests) were conducted.

**Results:**

In the last 3 years, 41% of the 78 respondents had practiced SMA. A higher percentage of those who believed that antibiotics are used to treat viral infections have self-treated compared to those who did not hold that belief. Of those who SMA, convenience was the most common reason, while the common symptoms treated were congestion and fever.

**Implications:**

The current study provides a first estimate of SMA in the Central Appalachian Region and finds the prevalence to be higher than previously reported in other regions of the U.S. Future studies could include larger, more representative samples and longitudinal study designs to confirm these findings.

## INTRODUCTION

Antimicrobial resistance (AMR) is “one of the biggest threats to global health…today.”^1^ It occurs when microbes—including bacteria, viruses, fungi, and parasites—adapt and grow in the presence of antimicrobial compounds that once impacted them.^2^ Annually, AMR accounts for more than 2.8 million infections. It results in more than 35,000 deaths in the U.S., around 700,000 deaths globally, and an added $20 billion in excess direct costs for the American healthcare system.^2^,^3^ Furthermore, AMR is estimated to cost Americans an additional $35 billion per year in lost productivity.^2^

Misuse and overuse of antimicrobials, including self-medication with antibiotics (SMA) can lead to AMR.^4^ SMA is defined by the World Health Organization (WHO) as “the acquisition of antibiotics and self-administering them (or administering them to a child), with the aim of treating a perceived infection, and intermittent or continued use of the prescribed drug for chronic or recurrent diseases without the advice of a qualified health professional.”^5^ It has been named one of the primary contributors to AMR that can be addressed by education and policy.^3^

SMA is a widespread issue globally, both in countries where antibiotics are prescription-only and where they are legally available over the counter.^6^–^9^ In a recent systematic review, Auta et al. estimated that 60–75+% of requests for over-the-counter antibiotics were filled in pharmacies.^7^ Self-medication and non-prescription antibiotic use is reported at rates ranging from 3% to 66% in the U.S.^4^ Data in the U.S. are limited in scope, as the majority come from specific population subgroups, primarily Latinx groups.^10^–^16^

SMA is associated with increasing rates of AMR, shorter and less-effective treatment courses, inappropriate drug and dose choice, adverse reactions, drug interactions, delayed hospital admissions, masking signs of disease, and incorrect or delayed diagnosis when medical care is finally sought.^7^,^17^,^18^ Many people are uninformed of the risks of SMA and subsequent AMR.^19^

Increased incidence of self-medication with antibiotics has been attributed to predisposing factors, like low levels of antimicrobial literacy (knowledge); enabling factors, such as accessibility of non-prescription antibiotics (leftovers or other source); and barriers to health care in a multitude of countries and settings.^4^,^19^–^21^ People in a European study who self-medicated with antibiotics were roughly twice as likely to think them appropriate for bronchitis or other minor ailments, live in an area with a lower gross domestic product, be middle aged or younger, have difficulty accessing healthcare, lack knowledge about antibiotics, and have financial constraints.^20^,^21^ Little is reported on predisposing factors in the U.S.^4^

SMA has not been assessed in Appalachia, though the risk factors and social determinants of health previously identified with SMA are prevalent in the region. To fill this gap, this study aims to (1) assess factors associated with SMA; (2) assess demographic differences in knowledge and beliefs of SMA; and (3) describe antimicrobial practices and beliefs of those who self-medicated in Claiborne County, Tennessee. Claiborne, as part of the Central Appalachian Region, lags behind much of the U.S., with a higher poverty rate (28%), lower rates of education, lower median household income (<$30,000), and lower number of physicians per capita (<40 per 100,000).^22^ Appalachia as a whole also has high rates of antibiotic prescribing, facilitating access to leftover prescriptions.^23^

## METHODS

### Sampling, Setting, and Procedure

Face-to-face structured interviews were conducted between October 2020 and March 2021 in the waiting rooms of a rural health clinic and dental office in Claiborne County, Tennessee. The state-subsidized, faith-based rural clinic serves primarily uninsured and underinsured patients from the rural county. The dental office serves a broader population of the same county. Interviews were conducted via convenience sampling in waiting areas of the offices. Interviewers directly approached the participants to introduce the study and asked for interest in participation. Face-to-face interviews allowed limited-literacy respondents to participate in the study by allowing researchers to clarify questions. Informed consent was verbally obtained prior to beginning the interview. The interviewer marked responses on the interview instrument during the conversations, which lasted between 5 and 15 minutes each. This study was approved by the Lincoln Memorial University Institutional Review Board (IRB # 954.v.1).

### Participants

Participants were eligible for inclusion if they presented in the waiting rooms, were over 18 years old, verbally consented, and had sufficient time to complete the interview. A sample size of 380 persons was calculated (total population of the county ~32,000, 95% CI, *p*<.05) using Select Statistical Services. Interviewers engaged 80 potential participants and completed 78 interviews. (One participant was called to their appointment and unable to complete the interview, so those results were not included; an additional person declined to participate.) The sampled population was smaller than the calculated size desired due to lack of patient visits in the offices during the sampling periods.

### Instrument

The survey instrument was adapted by the authors from a previously tested and standardized questionnaire.^18^ Additional questions were developed by the authors in consultation with public health faculty at East Tennessee State University. The instrument was pretested with a convenience sample of individuals with various levels of education and ages. The 33 questions covered demographics, knowledge of appropriate antibiotic use, and behaviors associated with self-medication.

### Data Processing

Stata version 17.0 was used for all statistical analyses (College Station TX). Questions that related to knowledge of common adverse symptoms of antibiotics were combined into a scale using the ‘alpha’ command. The scale was deemed acceptable if *α* ≥ 0.70. Any missing items of the scale were inputted using the mean. Inferential and descriptive analyses were conducted. Chi-squared and analysis of variance (ANOVA) tests were used to analyze differences in categorical and continuous variables, respectively. Fisher’s exact test was used in place of chi-squared if expected counts were less than 5. Normality and heteroskedasticity of residuals were checked when using ANOVA.

## RESULTS

### Study Sample

Due to COVID-19 restrictions in clinical waiting areas, only 78 complete responses were able to be collected from a mixed-gender population ranging in age from 18 to 80 years. This represented a participation rate of 99% of those who were approached for the interview. Forty-one percent of respondents had practiced SMA in the last three years. Knowledge of the different side effects of antibiotics was combined into a scale (*α* = 0.85), which had a mean and standard deviation of 0.60 and 0.37, respectively. The higher the number on the scale, the more frequently adverse symptoms were correctly identified.

### Objective 1. Factors Associated With SMA

No demographics were associated with SMA (Table 1). The only knowledge variable associated with SMA was the belief that antibiotics are used to treat viruses. A higher percentage of those who believe this (78.1%) had self-treated compared to those who do not (21.9%). There was little difference in SMA among groups with varying levels of education.

### Objective 2. Demographic Differences in Antibiotic Knowledge

There were no significant differences in antibiotic knowledge by demographic group (Table 2). Similar to Objective 1, there was no significant difference between groups with varying formal education levels (HS or less and at least some college) in regards to antibiotic knowledge.

### Objective 3. Practices and Beliefs of Those Who Self-medicated

Among those who self-medicated, the most common reason given was convenience (67.7%) (Table 3). The most common symptoms treated were congestion (38.2%) and fever (38.2%). The most common reason for selection of an AB was based on a previous prescription (79.4%). The majority who participated in self-medication believed that self-mediation is an acceptable practice (55.9%), and nearly half (47.1%) believed that they can successfully self-treat an infectious disease using AB.

## DISCUSSION

In this sample, the prevalence of SMA was 41%, higher than demonstrated in many other studies of various U.S. populations.^4^ The social determinants of health among the sample population are similar to those of other areas in rural Appalachia in that roughly half of those surveyed were uninsured or on Medicare/Medicaid, had no college education (12.82% did not graduate high school), and the plurality had annual household incomes under $25,000 (41.56%).^24^ The health clinic that was the site of interviews is the only care provider for within a 10-mile radius. These predisposing factors to SMA, coupled with few primary care physicians in the Central Appalachian Region (<40/100,000), may lead to higher than expected rates of self-medication with antibiotics.^19^–^21^,^24^ This in turn could cause poor health outcomes due to delayed appropriate treatment and increased likelihood of AMR.

The participants’ level of formal education did not significantly impact SMA or knowledge of antibiotics. Of the total population, 59% believed that antibiotics are used against viral infections. This was significantly higher than in a recent study by Zhang et al. in which 28% had a similar misconception.^25^ The incorrect assumption that antibiotics are used to treat viral infections was associated with a statistically significant tendency to SMA in our population and in other reports.^25^ When those who SMA reported what symptoms they commonly treated, the vast majority mentioned a cluster of symptoms (congestion, cough, sore throat, fever) that are commonly associated with viral respiratory diseases. It is unknown whether the participants understand the difference in viral and bacterial etiologies that present with similar symptoms. This aligns with other reports that people who self-medicate with antibiotics are roughly twice as likely to believe they are appropriate for bronchitis or other minor ailments.^20^,^21^

Availability of antibiotics is another reported predisposing factor.^19^,^25^ Antibiotics are prescription-only in the U.S., except for when bought in feed stores for certain agricultural applications. While this limits availability, it does not curtail SMA completely. Respondents who SMA predominantly reported using leftovers from a previous prescription (own or friend’s, 92%). Appalachia has high rates of antibiotic prescribing, which facilitates access to leftover prescriptions.^23^ Leftover antibiotics imply that the previous prescription was not completed as labelled. Not using the complete prescription as labelled (stopping treatment or skipping doses) can increase the risk of incomplete disease resolution and AMR.

The perception of SMA among those who practiced it varied. While the majority thought it was a good or acceptable practice (82.4%), only 47.1% were confident that they could themselves treat infectious diseases with antibiotics. This could be related to the inefficacy of treatment resulting from attempted treatment of viral infections, shortened duration of treatment, or inappropriate choice of antibiotic.

The impact of antimicrobial resistance and self-medication with antibiotics is felt by individuals and communities. Delayed diagnosis and treatment due to delays in seeking care or masking of symptoms by antibiotics can have significant deleterious effects. Physicians in Claiborne County report frequent cases of advanced metastatic cancers that were self-treated with antibiotics for months before seeking care.^26^ This can be devastating to the individual and their families when a grave prognosis might be avoided by earlier diagnosis.

### Limitations

Limitations of this study include a relatively small sample size due to the timing of the interviews coinciding with COVID-19 outbreak. Due to pandemic-related restrictions, patients were seen one at a time with little to no time spent in the waiting area. With a sample size of 78, the true estimated confidence level is approximately 62%, rather than the preferred 95%. The study is further limited by its use of convenience sampling at healthcare facilities. The sample may not be representative of the underlying population, especially of those without access to health care. The interview was also subject to recall bias, since the participants were asked about past use of antibiotics and may have difficulty remembering details.

## IMPLICATIONS

The current study provides a first estimate of SMA in part of Appalachia and finds that prevalence to be higher than previously reported among other U.S. subpopulations. Future studies could include larger, more representative samples and longitudinal study designs to confirm these findings.

SMA was frequently practiced among participants, potentially due to misunderstandings of what antibiotics treat (virus) and the convenience of treating with leftover antibiotics. Understanding the predisposing factors can guide educational interventions to decrease inappropriate use. The authors have developed an evidence-based, four-session, intrapersonal health education program designed to increase awareness of AMR and improve antibiotic stewardship based on the Precaution Adoption Process and Health Belief Models. This will be implemented within middle school science classrooms to improve beliefs about what antibiotics treat (to address the misconception discovered about virus treatment), improve prescription compliance, and foster self-efficacy in antimicrobial stewardship.

**SUMMARY BOX**

**What is already known about this topic?**

Self-medication with antibiotics is a prevalent practice worldwide. In the U.S., most studies have focused on immigrants. Prevalence rates in the U.S. have been reported from 3% to 66%.

**What is added by this report?**

This report provides a first estimate of SMA in part of Appalachia and finds the prevalence to be higher than previously reported in much of the U.S.— at 41%. It further explores the social determinants of health and knowledge, beliefs, and attitudes that contribute to self-medication with antibiotics.

**What are the implications for future research?**

Research in this field should continue to collect a more representative sample of this population and expand to other sub-populations. This could be used to develop health education interventions appropriate to the risk factors and knowledge of the target populations.

## Figures and Tables

**Table 1:** Associations of demographics and knowledge about antibiotics with SMA (n = 78)

	Have self-medicated (n = 32)	Have *not* self-medicated (n = 46)	Row total (n = 78)	*p*-value
**Age in years**, n(%)				
<40	7 (21.9)	12 (26.1)	19 (24.4)	0.29
40 to <60	16 (50.0)	15 (32.6)	31 (39.7)	
60 and older	9 (28.1)	19 (41.3)	28 (35.9)	
**Gender**, n(%)				
Male	13 (40.6)	18 (39.1)	31 (39.7)	0.89
Female	19 (59.4)	28 (60.9)	47 (60.3)	
**Education**, n(%)				
HS or less	15 (46.9)	24 (52.2)	39 (50.0)	0.65
At least some college	17 (53.1)	22 (47.8)	39 (50.0)	
**Income**, n(%)*				
<$25,000	15 (48.4)	17 (37.8)	32 (42.1)	0.43
$25,000–$49,000	8 (25.8)	10 (22.2)	18 (23.7)	
$50,000 and greater	8 (25.8)	18 (40.0)	26 (34.2)	
**Insurance**, n(%)				
Medicaid, Medicare, or none	19 (59.4)	23 (50.0)	42 (53.9)	0.42
Private, employer, or other	13 (40.6)	23 (50.0)	36 (46.2)	
**Know what antibiotics are**, n(%)†				
Yes	30 (93.8)	39 (84.8)	69 (88.5)	0.3
No	2 (6.3)	7 (15.2)	9 (11.5)	
**Believe antibiotics are for viruses**, n(%)				
Yes	7 (21.9)	24 (52.2)	31 (39.7)	0.01
No	25 (78.1)	22 (47.8)	47 (60.3)	
**Believe antibiotics are for bacteria**, n(%)				
Yes	24 (77.4)	32 (69.6)	56 (72.7)	0.45
No	7 (22.6)	14 (30.4)	21 (27.3)	

NOTES:

* Some participants declined to report; column totals different than total expected.

† Fisher’s exact test was used due to <5 expected counts.

**Table 2:** Demographic differences in antibiotic (AB) knowledge

	Know what antibiotics are		Believe that antibiotics are for viruses		Believe that antibiotics are for bacteria		Knowledge of adverse symptoms of AB scale	
**Age in years**, n(%)	n(%)	*p-*value*	n (%)	*p-*value	n (%)	*p-*value	Mean (sd)	*p-*value
<40	15 (79.0)	0.35	9 (47.4)	0.71	14 (73.7)	0.59	0.64 (0.37)	0.64
40-<60	28 (90.3)	-	11 (35.5)	-	20 (66.7)	-	0.55 (0.37)	-
60 and older	26 (92.9)	-	11 (39.3)	-	22 (78.6)	-	0.62 (0.36)	-
**Gender**, n(%)								
Male	28 (90.3)	1.0	9 (29.0)	0.12	26 (83.9)	0.07	0.59 (0.37)	0.93
Female	41 (87.2)	-	22 (46.8)	-	30 (65.22)	-	0.60 (0.37)	-
**Education**, n(%)								
HS or less	34 (87.2)	1.0	16 (41.0)	0.82	31 (79.5)	0.18	0.65 (0.37)	0.2
At least some college	35 (89.7)	-	15 (38.5)	-	25 (65.8)	-	0.54 (0.36)	-
**Income**, n(%)								
<$25,000	26 (81.3)	0.23	11 (34.4)	0.62	25 (78.1)	0.43^1^	0.63 (0.40)	0.5
$25,000-$49,000	16 (88.9)	-	8 (44.4)	-	14 (77.8)	-	0.50 (0.32)	-
$50,000 and greater	25 (96.2)	-	12 (38.7)	-	16 (64.0)	-	0.61 (0.36)	-
**Insurance**, n(%)								
Medicaid, Medicare, or none	37 (88.1)	1.0	16 (38.1)	0.75	32 (76.2)	0.46	0.60 (0.36)	0.86
Private, employer, or other	32 (88.9)	-	15 (41.7)	-	24 (68.6)	0.59 (0.38)	-	

NOTE:

* Fisher’s exact test was used due to <5 expected counts.

**Table 3:** Practices and beliefs of those who self-medicated (n = 34)

	n (%)
**Times treated in last year**	
0	6 (17.8)
1	16 (47.1)
2 or more	12 (35.3)
**Reasons for SMA** *	
Cost	13 (38.2)
Convenience	23 (67.7)
Lack of trust	3 (8.8)
**Symptoms treated by SMA** *	
Rhinorrhea	9 (26.5)
Congestion	13 (38.2)
Cough	11 (32.4)
Sore throat	11 (32.4)
Fever	13 (38.2)
Aches	3 (8.8)
Vomiting	1 (2.9)
Diarrhea	0 (0.0)
Skin	5 (14.7)
Dental	4 (12.9)
**Selection of AB based on…** *	
Pharmacy	2 (5.9)
Family members	1 (2.9)
Friends	1 (2.9)
Own experience	8 (23.5)
Internet	1 (2.9)
Previous prescription	27 (79.4)
Advertisement	0 (0.0)
**Obtained AB from…** *	
Pharmacy	6 (17.7)
Leftover from own rx	26 (74.5)
Leftover from friend/family rx	6 (17.7)
Online	0 (0.0)
Vet/feed store	2 (5.9)
**Chose dose of AB based on…** *	
Package insert	8 (24.2)
Doctor	10 (29.4)
Pharmacist	5 (14.7)
Family or friends	1 (2.9)
Media	1 (2.9)
Internet	2 (5.9)
Experience	15 (44.1)
Guess	5 (14.7)
**Perception of SMA**	
Not acceptable	6 (17.7)
Acceptable practice	19 (55.9)
Good practice	9 (26.5)
**Can treat infectious diseases with AB?**	
Yes, I can	16 (47.1)
No, I cannot	8 (23.5)
Not sure	10 (29.4)

NOTE:

* Multi-select questions; percentages will not add up to 100.
